# Neck-Tongue Syndrome: Viewpoints on Etiology in a Patient with Bilateral Symptoms

**DOI:** 10.1155/2018/9131068

**Published:** 2018-05-31

**Authors:** Jonathan S. Sidlow, Mark J. Raden, Richard Sidlow

**Affiliations:** ^1^Division of Pediatric Hospital Medicine, Department of Pediatrics, Staten Island University Hospital-Northwell Health, Staten Island, New York, USA; ^2^Department of Radiology, Staten Island University Hospital-Northwell Health, Staten Island, New York, USA

## Abstract

Neck-Tongue Syndrome is a rare entity, and when it presents in the pediatric age group, it is usually due to osseous, ligamentous, or nervous anatomic variation. We present below a case involving a patient whose bilateral symptoms were intermittently present from the age of five to the age of twenty-one years and discuss this case in light of the present theories of the anatomic substrate underlying this syndrome.

## 1. Introduction

Neck-Tongue Syndrome (NTS) is a type of cervicogenic headache recently reviewed by Gelfand et al. whose proposed criteria entail unilateral neck and/or occipital pain accompanied by ipsilateral tongue dysesthesia and/or abnormal posture upon sudden turning of the neck. [1] These symptoms can last anywhere from 10 seconds to several minutes in duration and can occasionally be accompanied by various other symptoms involving the oropharynx and upper extremities [1]. The etiology of childhood/adolescent NTS, unlike adult NTS which is most often due to trauma or inflammatory diseases involving the cervical spine, remains unclear but invokes variations in osseous/ligamentous and nervous anatomy to explain this uncommon nexus of symptomology [2]. Given the rarity of this syndrome, however, no one explanation has proven to be all encompassing. We describe below a case of this syndrome which began in a patient at five years of age and persisted into adulthood and then present a discussion of the different etiologic theories proposed for NTS in its light.

## 2. Case Report

A five-year-old male began experiencing a popping sensation followed immediately by the sensation of heat and pressure starting medially at the base of the skull which radiated two centimeters bilaterally. He was a product of a nonconsanguineous union, whose birth history did not involve instrumentation and whose past medical, surgical, and developmental histories were all noncontributory. This sensation was experienced simultaneously with bilateral numbness of the posterior tongue and difficulty in speaking, both of which lasted fifteen seconds with gradual attenuation. The above would occur upon abrupt lateral rotation (either direction) of the head approximately five to ten times a year unrelated to trauma, diminishing in frequency as the patient aged.

Between the ages of eleven and fifteen, as a competitive fencer, the patient would experience the same symptomology when performing actions involving extreme, abrupt lateral rotation of the head, translating to a frequency of approximately once to twice weekly.

At the age of nineteen, during military training and combat, the same symptomology was experienced approximately fifteen times when abrupt lateral rotation of the head prompted by various forms of minor external head trauma was experienced. After discharge from the military, frequency of these episodes was sustained with simple lateral rotation of the head outside the context of trauma for six months with spontaneous abatement.

**Figure 1:**
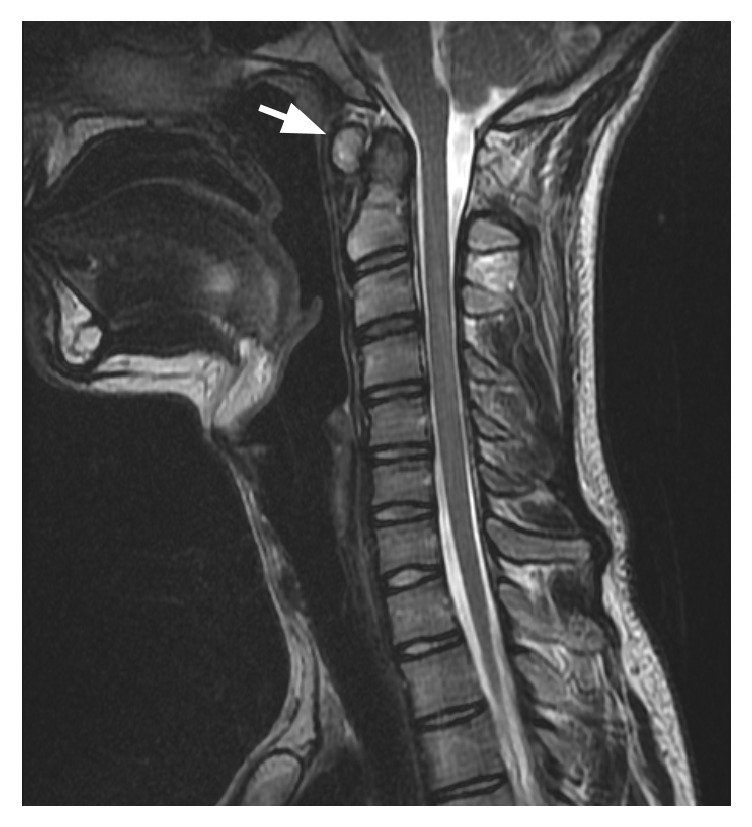
Cervical MRI image revealing slight dysplastic enlargement of the anterior arch of C1 vertebrae (white arrow).

**Figure 2:**
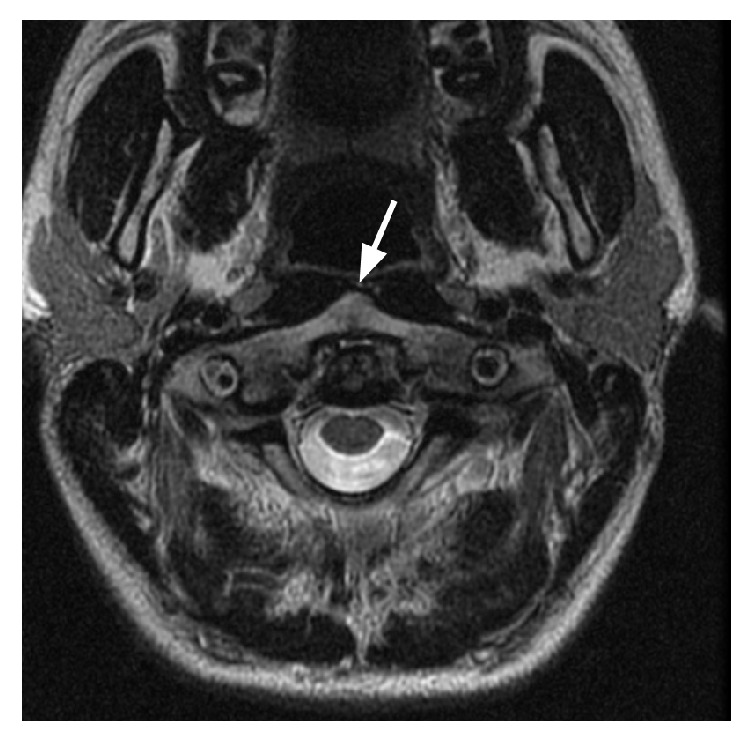
Cervical MRI image showing mild degenerative changes of the atlantoaxial junction (white arrow).

**Figure 3:**
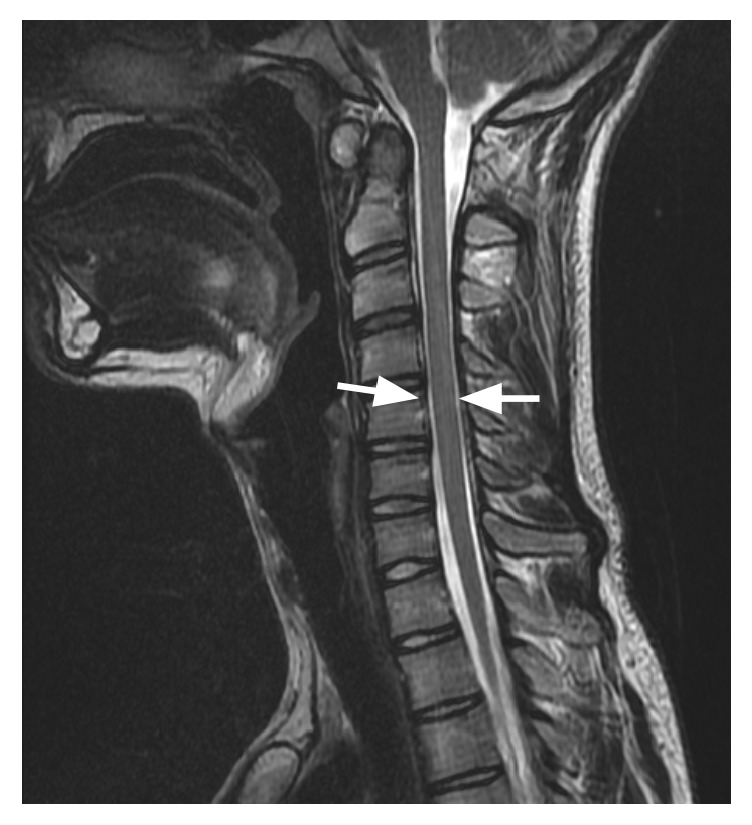
Cervical MRI image showing spinal canal caliber on the lower end of normal limits (white arrows).

At the age of twenty-one, the first time the patient came to medical attention for the above complaint, the patient's physical and neurologic examinations were completely normal, including cranial nerve exam. Of note, the patient was not hyperelastic, nor was there a family history of the same. Magnetic resonance imaging (MRI) of the cervical spine (Figures 1–3) was performed revealing slight dysplastic enlargement of the anterior arch of C1 vertebrate, mild degenerative changes of the atlantoaxial junction, and spinal canal caliber on the lower end of normal limits. No evidence for abnormal bone spurring or abnormality of the hypoglossal canal was found.

Of note, upon further query, it was found that the patient's grandmother also had the same condition.

## 3. Discussion

In comparison to Gelfand et al.'s recent review, two aspects of this case are of note. Firstly, no cases of midline neck/occipital pain and only one case of bilateral tongue symptoms were reported in the review [1, 3]. Bogduk, adding to Lance and Anthony's contributions to the anatomic explanation for this syndrome, argued that proprioceptive fibers from the lingual nerve returning via the C2 ventral ramus/cervical plexus are impacted against the edge of an articular process of the atlantoaxial joint. Additionally, Bogduk believed that the ipsilateral neck pain component of this syndrome is caused by abnormal subluxation of that joint rather than by nerve compression because experimental compression of spinal nerves or peripheral nerves has produced numbness but not pain [4, 5]. In light of the osseous and ligamentous upper cervical findings found on MRI in this patient, these theories are supported and could explain the midline/bilateral symptomology experienced by him.

Secondly, the temporal pattern of symptomology exhibited by this patient argued that the modicum of ligamentous laxity of his atlantoaxial joint was not increasing over time, but rather decreasing, as evidenced by increasing lateral rotational forces needed to elicit symptoms. It is possible that despite this patient's comparatively small spinal canal any component of ligamentous laxity may have been overridden by proportionally normal growth of the bony and ligamentous structures of his cervical spine. As a result, on an absolute level, increased room for and less traction on the ventral ramus of C2 within the spinal canal over time necessitated more ballistic movements involving a larger rotational excursion to elicit symptoms as the patient aged.

Cassidy et al. suggest that entrapment of the ventral ramus of C2 nerve could be achieved by spasm of the suboccipital muscles, especially the inferior oblique muscle, because it exits beneath that muscle and is attached to it through fascial attachments [6]. While this theory could provide an anatomic explanation for the bilaterality of symptoms and their spontaneous resolution, it does not explain the diminution in frequency and increased force needed over time to elicit symptomology in our patient.

Wong et al. take a broader approach to the anatomic variations underlying NTS [7]. In theorizing about a common cause of NTS symptomology in a 14-year-old girl subsequently found to have a Chiari-1 malformation with 10 mm tonsillar descent, they cite a 30-50% overlap of anomalies of the base of the skull and spine in patients with Chiari-1 malformations. These anomalies include cervical spina bifida occulta, cervical scoliosis, Klippel-Feil deformity, atlantoaxial assimilation, atlantooccipital fusion, and basilar invagination. They suggest that given this association the etiologies of NTS and Chiari-1 malformations are probably the same, namely, developmental variation along a spectrum of severity common to the base of the skull and upper cervical spine.

Common mechanisms for Chiari malformations and Klippel-Feil syndrome have been previously proposed; i.e., defects of postotic neural crest (PONC) cells and subclavian artery supply disruption sequence (SASDS) have been hypothesized as common explanations for Klippel-Feil syndrome and Moebius syndrome [8, 9]. Given the above, it may be possible to argue that NTS is a mild variant of SASDS or PONC migration, both mechanisms providing reasonable explanations for combined musculoskeletal and nervous anatomic variation. Additional support for a common etiology of NTS and other malformations of the base of the skull and cervical spine exists based on the existence of a possible genetic link between Chiari-1 malformations and Klippel-Feil syndrome in patients without collagenopathies involving mutations in the genes GDF3 and GDF6 which code for osseous growth differentiation factors [10].

## 4. Conclusion

NTS remains an intriguing syndrome whose theorized combined musculoskeletal and nervous etiologies argue a common embryologic origin. Our case, atypical for its bilateral symptomology and its resolution over developmental time, lends credence to ligamentous laxity playing a role, at least initially, in the emergence of our patient's symptoms but does not explain its natural history. More research, both clinical and genetic, is necessary to define whether NTS is an entity unto itself or part of a larger spectrum of developmental variation of the base of the skull and cervical spine.
